# Supplementary material for: Comparing the influence of stimulus size and contrast on the perception of moving gratings and random dot patterns—A registered report protocol
Source: PLoS One. 2021 Jun 21;16(6):e0253067. doi: 10.1371/journal.pone.0253067 (PMC8216547; doi:10.1371/journal.pone.0253067)
Supplement: S1 Appendix — (PDF) [file pone.0253067.s001.pdf]

S1 – Appendix: Results of pilot experiment

Methods summary

We tested how stimulus size and contrast affect the perception of direction of moving sinusoidal gratings (“Gabor patches”) compared to random dot patterns (RDPs) as measured by the percentage of correct choices in a direction discrimination task.

The results described here were collected from 15 right-handed subjects (9 female, 6 male; ages 20-34 years, mean age = 24.1 years) with normal or corrected-to-normal vision.

The experimental setup was identical to the one described in the main text.

Subjects were presented with either a horizontally drifting, sinusoidal grating with a 2D Gaussian envelope (“Gabor patch”) or a random dot pattern (RDP) and had to report whether a stimulus was moving to the left or to the right.

The grating had a spatial frequency of 1 cycle per degree of visual angle. The radius of each grating was defined as twice the standard deviation of the Gaussian envelope (2σ). We used σ values of 0.4°, 1.0°, 1.6°, 2.2°, and 2.8°, resulting in gratings of radius 0.8°, 2.0°, 3.2°, 4.4°, and 5.6°. The phase of the grating at stimulus onset varied randomly from trial to trial and moved with a speed of 3.5°/s to the left or the right.

Three different contrast levels were presented in different blocks by changing the gratings’ transparency. Michelson contrasts, calculated as $\frac{I_{max}-I_{min}}{I_{max}+I_{min}}$ (where I_{max} is the highest luminance (i.e., the brightest white) and I_{min} is the lowest luminance (i.e., the darkest black) of the grating) for what we call the “high”, “intermediate”, and “low” contrast condition were 99.2% [I_{max}: 70 cd/m^2, I_{min}: 0.3 cd/m^2], 8.3% [I_{max}: 39 cd/m^2, I_{min}: 33 cd/m^2], and 2.8%. [I_{max}: 37 cd/m^2, I_{min}: 35 cd/m^2].

Random dot patterns had radii of 1°, 2.5°, 4°, 5.5°, and 7° (which, perceptually, resembled the size of our gratings) and a dot density of 4 dots/deg^2 (i.e., between 12 and 615 dots). All dots were placed randomly within the aperture and moved either to the left or to the right with a speed of 2 °/s. Again, three different contrast levels (I_{max} and I_{min}now being the luminance of the dots and of the background, respectively) were used in different blocks: 32% [I_{max}: 70 cd/m^2, I_{min}: 36 cd/m^2], 13.3% [I_{max}: 47cd/m^2, I_{min}: 36 cd/m^2], and 5.3% [I_{max}: 40 cd/m^2, I_{min}: 36 cd/m^2], termed “high”, “intermediate”, and “low” contrast, respectively.

Subjects were asked to foveate the center of the screen and started each trial with a button press. The fixation dot then disappeared and the stimulus was presented for a brief duration

that had been adjusted for each subject individually based on a training session (see below for further information). Subsequently, the stimulus was masked for 220ms with an RDP of radius 15° that had 2,000 black and 2,000 white dots (density: 5.7 dots/deg^2), all moving in random directions at 15 deg/s. After the mask disappeared and the fixation point reappeared subjects reported their perceived direction (left vs. right) by pressing the corresponding trigger button on the gamepad.

The experiment comprised 18 blocks of 100 trials each: For each stimulus type (grating and RDP) and every contrast level (high, intermediate, low), subjects completed 3 blocks (300 trials). Within a block, every stimulus size was presented 20 times in random order. Thus, each subject saw every possible combination of a specific stimulus type, contrast, and size 60 times. Blocks with the same stimulus type and blocks with the same contrast level were presented after one another: the three blocks with a high-contrast grating were followed by three blocks of the intermediate-contrast and three blocks of the low-contrast gratings and then a total of nine blocks of high-, intermediate-, and low-contrast RDPs.

To familiarize subjects with the task, they underwent a training session that took place 1-7 days before the actual test session. Each subject practiced with at least 1,000 trials, using gratings and RDPs of varying sizes and contrast levels (though not necessarily the exact values that were used during the experiment). The presentation duration of the stimulus was varied during the training session, starting with long durations until subjects had understood the task well. Towards the end of the training session, one stimulus duration for gratings and one for RDPs of the highest-contrast level was determined manually for which the subjects achieved approximately 75% correct responses for a stimulus of the intermediate size of the 5 sizes used in the main experiment.

To investigate whether performance was affected by stimulus size and how this size-effect was influenced by contrast, we employed two analysis steps: First, we wanted to determine whether a subject's performance was influenced by stimulus size for a given combination of stimulus type and contrast level. For this purpose, we calculated Pearson's correlation coefficient between stimulus size and performance for each subject for each of the six combinations of stimulus type and contrast level. For each correlation coefficient we calculated a 95% confidence interval (CI) using a bootstrapping approach with 2000 bootstrap replicates. We considered a subject to show a "negative size effect" (i.e., performance decreases with increasing stimulus size) if the entire 95% CI was smaller than 0 and a "positive

size effect” (i.e., performance increases with increasing stimulus size) if the entire 95% CI was larger than 0. If a confidence interval crossed 0, we considered the subject not to show a significant size effect for that condition.

Second, we calculated a three-way repeated-measures analysis of variance (rmANOVA), with “stimulus type” (grating or RDP), “contrast” (“high”, “intermediate”, or “low”) and “stimulus size” as within-subject factors. Degrees of freedom were Greenhouse-Geisser corrected whenever the assumption of sphericity was violated as assessed by Mauchly’s test. The equality of variance across stimulus sizes was confirmed to be similar within each of the 6 conditions using Levene’s test.

Results

The average stimulus duration across subjects did not differ significantly between gratings (M = 78.5ms, SD = 33.71) and RDPs (M = 74.6ms, SD = 40.69) (paired t-test: $t(14) = 0.76$, $p = .46$, $d = 0.11$). We had manually determined a duration for each stimulus type for which observers should achieve a performance level of approximately 75% for high-contrast stimuli of the intermediate size. Indeed, one-sample *t*-tests confirmed that performance levels across all subjects did not differ significantly from 75% for the 4° high-contrast RDP ($t(14) = -0.17$, $p = .87$) or the 3.2° high-contrast grating ($t(14) = 1.32$, $p = .21$).

Figure 1 plots the results of a representative subject. For the grating (top row) the subject meets our criterion for a negative size effect for all three contrast levels: the correlation coefficient between stimulus size and performance is negative and the 95% confidence interval is entirely below 0. For the random dot patterns (bottom row) the subject shows no size effect for either contrast level according to our criteria: for all three cases, the 95% confidence interval crosses 0. Note also that the correlation coefficients are much lower than for the gratings.

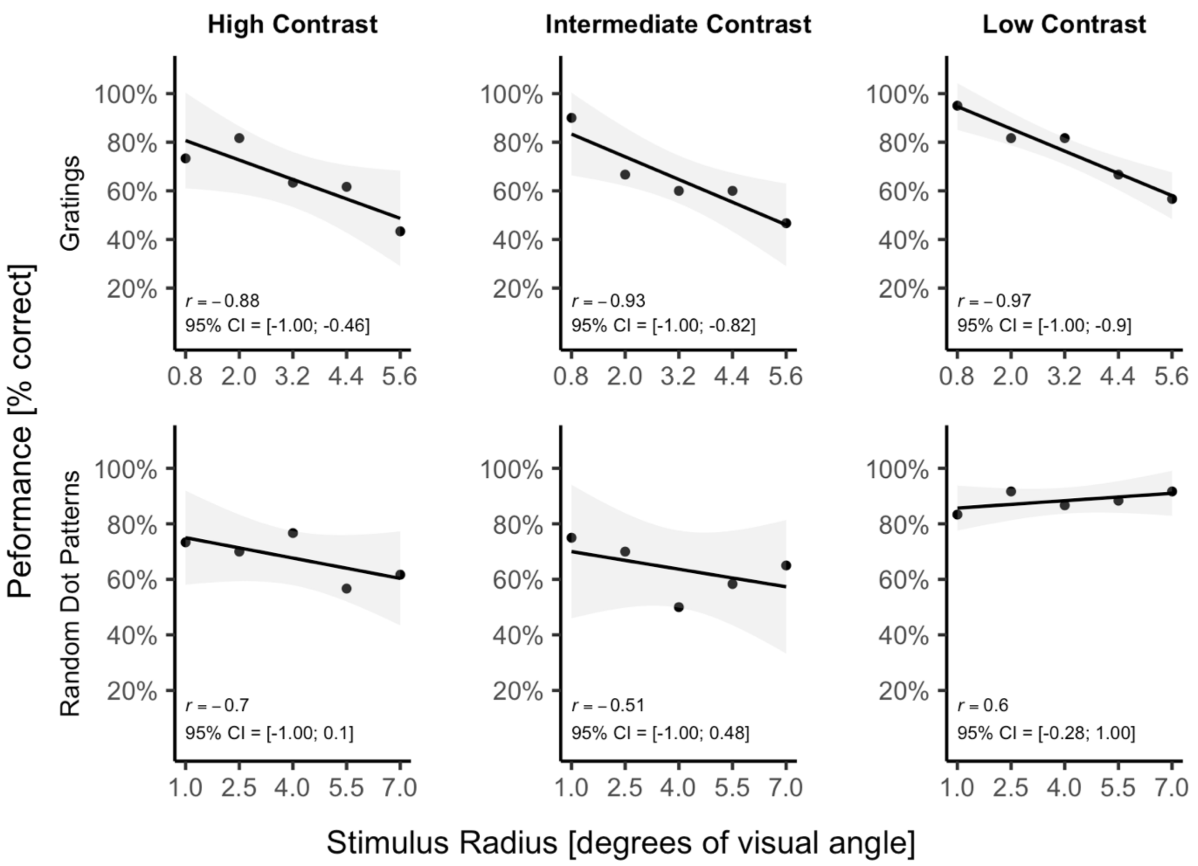

Figure 1: Effect of stimulus size on direction discrimination performance for an example subject in six different conditions: the top row shows data for the drifting grating, the bottom row for the random dot pattern. The three columns show data for three different contrast levels of the respective stimulus. This subject fulfilled our criteria for a negative size effect (negative correlation with a 95% confidence interval that lies entirely below 0) for all contrast levels for the grating, but not for any contrast level for the random dot pattern. Pearson's correlation coefficient and the 95% confidence interval are shown in each graph.

Across our 15 subjects, at least half show a negative size effect for each of the three contrast levels of the grating and none shows a positive size effect (Fig. 2). For RDPs, on the other hand, less than a third show a negative size effect and most subjects show no size effect at all

(Fig. 3).

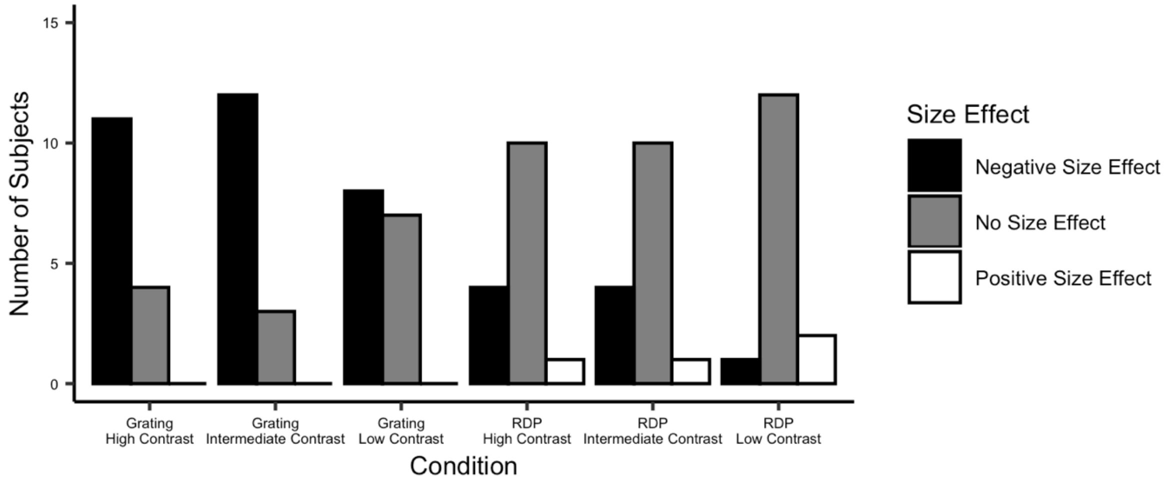

Figure 2: The number of subjects that showed a negative size effect (black bars), no size effect (gray bars) or a positive size effect (white bars) in each of the six conditions. See Methods for a definition of size effect.

There is a significant main effects of "stimulus type", based on a three-way repeated measures ANOVA, $F(1, 14) = 11.86$, $p < .01$, and "stimulus size", $F(4, 56) = 30.83$, $p < .001$, but no main effect of "contrast", $F(1.34, 18.75) = 0.41$, $p > .05$.
The interactions between "stimulus type" and "stimulus size" ($F(2.04, 28.63) = 20.75$, $p < .01$) and between "contrast" and "stimulus size" ($F(2.76, 38.70) = 3.90$, $p < .05$) are significant, but neither the interaction between "stimulus type" and "contrast" ($F(2, 28) = 1.64$, $p > .05$) nor the three-way interaction between "stimulus type", "contrast", and "stimulus size" ($F(2.63, 36.80) = 1.74$, $p>0.1$). In other words, the effect of stimulus size depends on the stimulus type. This can be clearly seen in the averaged data of all subjects for all six conditions, which show impaired performance with increasing stimulus size across all contrast levels for gratings (Fig. 3 top row), but not random dot patterns (Fig 3., bottom row).

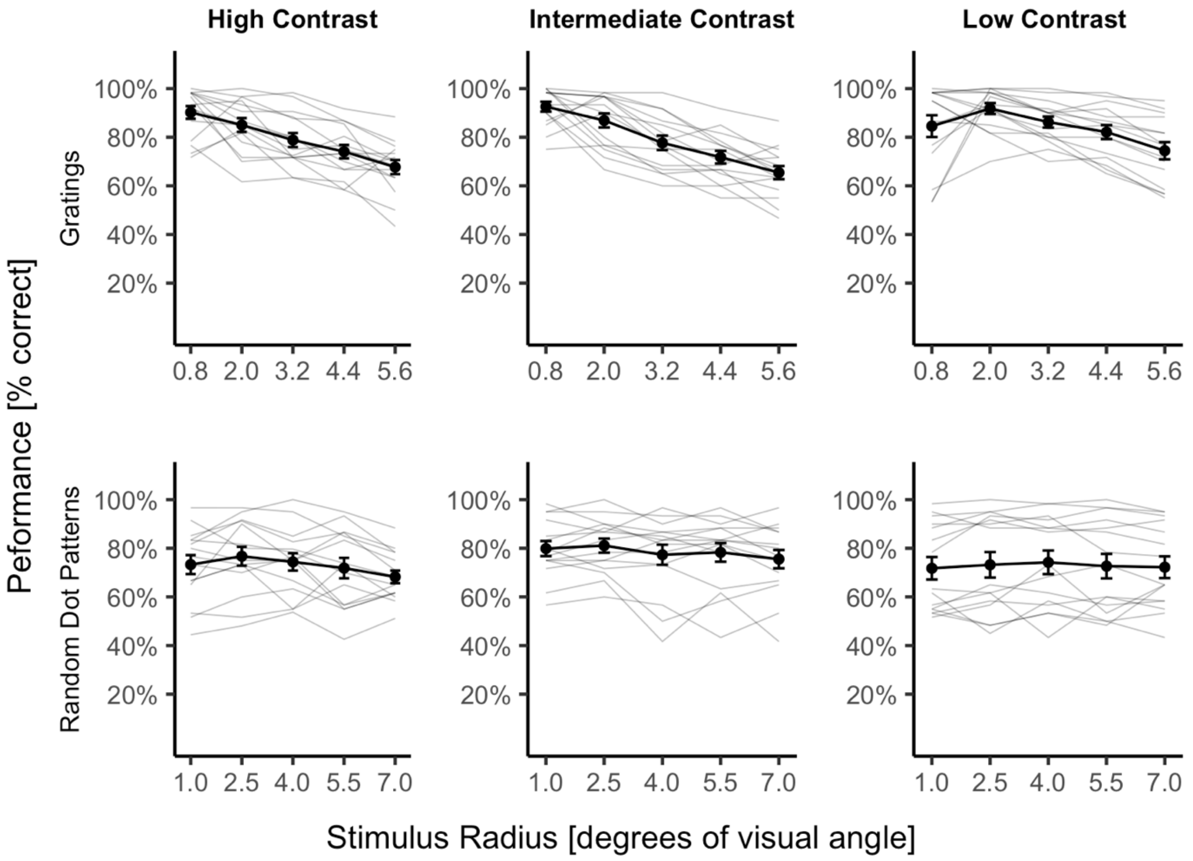

Figure 3: Effect of stimulus size on direction discrimination performance across all 15 subjects. The large black dots and lines show the mean across subjects, the thin gray lines show data from individual subjects, error bars show standard error of the mean. All other information is depicted as in Fig. 2.

Discussion

Our study compared the effects of size and contrast on how well the direction of motion of those two stimulus types can be discriminated. We observed two surprising differences compared to previous studies: first, while we were able to replicate the effect of stimulus size on performance for high-contrast gratings, we observed no such effect for random dot patterns. Second, we could only replicate the finding that performance decreases with increasing stimulus size for high-contrast gratings, but we did not observe the reverse effect for low contrast gratings that had previously been described (1).

There are a few, potentially important weaknesses of the experiment that could potentially explain some of the differences between previous studies (1–3) and ours:

First, the use of a mask right after the moving stimuli: visual persistence, i.e., the extended perception of a visual stimulus after the stimulus has been terminated, has been shown to be inversely related to stimulus duration and luminance (4). Therefore, it is likely that visual persistence varied across the different contrast-levels, stimulus sizes, and stimulus durations of other and our studies.

Second, we presented the different stimulus types and contrast levels in a blocked design with the order of blocks being identical across subjects. Thus, differences between gratings and RDPs (and potentially between different contrast levels) could be due to training effects.

Third, our two stimulus types (gratings and RDPs) differed in a number of features, such as speed and the overall luminance (which is kept at the level of the background for sinusoidal gratings, but increases above the background for RDPs with white dots).

We address these three points in our proposal by (1) using a mask on only half of the trials, (2) completely randomizing the presentation of all possible stimuli (i.e., all combinations of stimulus type, size, contrast, and the presence or absence of a mask), and (3) making RDPs and gratings more similar by using equal speeds for both stimuli and using RDPs that have 50% white and 50% black dots, thus ensuring stable luminance (see main text).

References

1. Tadin D, Lappin JS, Gilroy LA, Blake R. Perceptual consequences of centre – surround antagonism in visual motion processing. Nature. 2003;424:312–5.
2. Yazdani P, Serrano-Pedraza I, Whittaker RG, Trevelyan AJ, Read JCA. Two common psychophysical measures of surround suppression reflect independent neuronal mechanisms. J Vis. 2015;15(13):1–14.
3. Serrano-Pedraza I, Hogg EL, Read JCA. Spatial non-homogeneity of the antagonistic surround in motion perception. J Vis. 2011;11(2):1–9.
4. Bowen RW, Pola J, Matin L. Visual persistence: Effects of flash luminance, duration and energy. Vision Res. 1974;14(4):295–303.
5. Tadin D, Lappin JS. Optimal size for perceiving motion decreases with contrast. Vision Res. 2005;45(16):2059–64.
6. Robson JG. Spatial and Temporal Contrast-Sensitivity Functions of the Visual System. J Opt Soc Am. 1966;56(8):1141–2.
