# Supplementary material for: Comparing the influence of stimulus size and contrast on the perception of moving gratings and random dot patterns—A registered report protocol
Source: PLoS One. 2021 Jun 21;16(6):e0253067. doi: 10.1371/journal.pone.0253067 (PMC8216547; doi:10.1371/journal.pone.0253067)
Supplement: S1 Dataset — (PDF) [file pone.0253067.s002.pdf]

Supporting information for Wild & Treue, Comparing the influence of stimulus size and contrast on the perception of moving gratings and random dot patterns

S1 Dataset from Pilot Experiment; highlighted subject's (feb) data is shown in Fig 1 of S1 Appendix						
**Subject**	**Condition**	**Size 1**	**Size 2**	**Size 3**	**Size 4**	**Size 5**
ahs	Grating - High	0.93	0.95	0.72	0.67	0.67
ark	Grating - High	0.77	0.62	0.63	0.58	0.50
ays	Grating - High	0.93	0.72	0.72	0.73	0.73
dvm	Grating - High	0.87	0.85	0.80	0.70	0.63
feb	Grating - High	0.73	0.82	0.63	0.62	0.43
her	Grating - High	0.72	0.83	0.72	0.58	0.75
hrs	Grating - High	0.98	1.00	0.97	0.87	0.78
jol	Grating - High	0.78	0.97	0.98	0.92	0.88
mgd	Grating - High	0.98	0.78	0.72	0.80	0.70
mrg	Grating - High	0.98	0.87	0.80	0.67	0.72
ped	Grating - High	0.98	0.93	0.88	0.75	0.68
tim	Grating - High	0.97	0.70	0.72	0.73	0.63
veb	Grating - High	1.00	0.97	0.88	0.87	0.76
wos	Grating - High	0.92	0.85	0.75	0.77	0.70
zon	Grating - High	0.98	0.91	0.91	0.85	0.57
ahs	Grating - Intermediate	0.98	0.97	0.92	0.78	0.65
ark	Grating - Intermediate	0.95	0.75	0.67	0.67	0.50
ays	Grating - Intermediate	1.00	0.77	0.68	0.68	0.77
dvm	Grating - Intermediate	0.93	0.93	0.75	0.75	0.63
feb	Grating - Intermediate	0.90	0.67	0.60	0.60	0.47
her	Grating - Intermediate	0.80	0.90	0.85	0.77	0.68
hrs	Grating - Intermediate	0.90	0.72	0.65	0.67	0.63
jol	Grating - Intermediate	0.85	0.98	0.98	0.92	0.87
mgd	Grating - Intermediate	1.00	0.80	0.65	0.55	0.55
mrg	Grating - Intermediate	0.98	0.97	0.82	0.70	0.72
ped	Grating - Intermediate	0.98	0.90	0.77	0.65	0.58
tim	Grating - Intermediate	0.98	0.97	0.87	0.82	0.75
veb	Grating - Intermediate	1.00	0.98	0.92	0.77	0.67
wos	Grating - Intermediate	0.87	0.97	0.78	0.85	0.72
zon	Grating - Intermediate	0.75	0.77	0.75	0.60	0.63
ahs	Grating - Low	0.73	0.98	0.95	0.88	0.88
ark	Grating - Low	0.95	0.82	0.70	0.72	0.58
ays	Grating - Low	0.98	0.90	0.83	0.85	0.82
dvm	Grating - Low	0.85	0.93	0.92	0.85	0.77
feb	Grating - Low	0.95	0.82	0.82	0.67	0.57
her	Grating - Low	0.58	0.70	0.75	0.68	0.55
hrs	Grating - Low	1.00	0.98	0.90	0.80	0.70
jol	Grating - Low	0.98	0.92	0.78	0.65	0.57
mgd	Grating - Low	0.77	0.92	0.80	0.80	0.72
mrg	Grating - Low	0.98	1.00	0.90	0.95	0.90
ped	Grating - Low	0.98	0.98	0.90	0.87	0.82
tim	Grating - Low	0.53	1.00	0.98	0.98	0.92
veb	Grating - Low	0.98	1.00	1.00	0.97	0.95
wos	Grating - Low	0.53	0.97	0.90	0.92	0.77
zon	Grating - Low	0.87	0.85	0.80	0.73	0.67

ahs	RDP - High	0.82	0.92	0.85	0.93	0.78
ark	RDP - High	0.53	0.52	0.55	0.65	0.58
ays	RDP - High	0.52	0.60	0.63	0.55	0.62
dvm	RDP - High	0.67	0.73	0.55	0.73	0.60
feb	RDP - High	0.73	0.70	0.77	0.57	0.62
her	RDP - High	0.83	0.82	0.78	0.70	0.65
hrs	RDP - High	0.83	0.95	1.00	0.95	0.88
jol	RDP - High	0.97	0.97	0.95	0.85	0.78
mgd	RDP - High	0.85	0.91	0.83	0.86	0.71
mrg	RDP - High	0.67	0.73	0.67	0.57	0.65
ped	RDP - High	0.92	0.80	0.80	0.74	0.68
tim	RDP - High	0.77	0.73	0.75	0.87	0.80
veb	RDP - High	0.80	0.75	0.75	0.55	0.62
wos	RDP - High	0.65	0.90	0.75	0.83	0.75
zon	RDP - High	0.44	0.48	0.54	0.43	0.51
ahs	RDP - Intermediate	0.57	0.60	0.57	0.43	0.53
ark	RDP - Intermediate	0.62	0.67	0.42	0.62	0.42
ays	RDP - Intermediate	0.77	0.83	0.87	0.83	0.82
dvm	RDP - Intermediate	0.80	0.72	0.73	0.82	0.70
feb	RDP - Intermediate	0.75	0.70	0.50	0.58	0.65
her	RDP - Intermediate	0.75	0.75	0.73	0.63	0.67
hrs	RDP - Intermediate	0.83	0.90	0.87	0.88	0.88
jol	RDP - Intermediate	0.95	1.00	0.90	0.97	0.87
mgd	RDP - Intermediate	0.78	0.88	0.83	0.88	0.77
mrg	RDP - Intermediate	0.92	0.87	0.97	0.90	0.97
ped	RDP - Intermediate	0.98	0.90	0.82	0.82	0.75
tim	RDP - Intermediate	0.85	0.87	0.85	0.78	0.75
veb	RDP - Intermediate	0.95	0.95	0.93	0.93	0.87
wos	RDP - Intermediate	0.75	0.78	0.78	0.83	0.90
zon	RDP - Intermediate	0.72	0.75	0.83	0.83	0.80
ahs	RDP - Low	0.62	0.45	0.58	0.50	0.43
ark	RDP - Low	0.95	0.88	0.88	0.87	0.82
ays	RDP - Low	0.93	0.95	0.98	0.97	0.95
dvm	RDP - Low	0.63	0.62	0.68	0.73	0.65
feb	RDP - Low	0.83	0.92	0.87	0.88	0.92
her	RDP - Low	0.55	0.48	0.53	0.50	0.53
hrs	RDP - Low	0.55	0.65	0.62	0.57	0.58
jol	RDP - Low	0.88	0.95	0.88	0.92	0.87
mgd	RDP - Low	0.53	0.58	0.57	0.60	0.58
mrg	RDP - Low	0.98	1.00	0.98	1.00	0.95
ped	RDP - Low	0.52	0.57	0.73	0.53	0.65
tim	RDP - Low	0.53	0.48	0.53	0.48	0.65
veb	RDP - Low	0.78	0.93	0.92	0.97	0.93
wos	RDP - Low	0.57	0.62	0.43	0.60	0.55
zon	RDP - Low	0.90	0.90	0.93	0.78	0.77
